# The electronic structure of FeV-cofactor in vanadium-dependent nitrogenase†

**DOI:** 10.1039/d0sc06561g

**Published:** 2021-03-29

**Authors:** Zhi-Yong Yang, Emilio Jimenez-Vicente, Hayden Kallas, Dmitriy A. Lukoyanov, Hao Yang, Julia S. Martin del Campo, Dennis R. Dean, Brian M. Hoffman, Lance C. Seefeldt

**Affiliations:** Department of Chemistry and Biochemistry, Utah State University Logan UT 84322 USA nkyzy@hotmail.com lance.seefeldt@usu.edu +1-435-797-3964; Department of Biochemistry, Virginia Tech Blacksburg VA 24061 USA deandr@vt.edu +1-540-231-5895; Department of Chemistry, Northwestern University Evanston IL 60208 USA bmh@northwestern.edu +1-847-491-3104

## Abstract

The electronic structure of the active-site metal cofactor (FeV-cofactor) of resting-state V-dependent nitrogenase has been an open question, with earlier studies indicating that it exhibits a broad *S* = 3/2 EPR signal (Kramers state) having *g* values of ∼4.3 and 3.8, along with suggestions that it contains metal-ions with valencies [1V^3+^, 3Fe^3+^, 4Fe^2+^]. In the present work, genetic, biochemical, and spectroscopic approaches were combined to reveal that the EPR signals previously assigned to FeV-cofactor do not correlate with active VFe-protein, and thus cannot arise from the resting-state of catalytically relevant FeV-cofactor. It, instead, appears resting-state FeV-cofactor is either diamagnetic, *S* = 0, or non-Kramers, integer-spin (*S* = 1, 2 *etc.*). When VFe-protein is freeze-trapped during high-flux turnover with its natural electron-donating partner Fe protein, conditions which populate reduced states of the FeV-cofactor, a new rhombic *S* = 1/2 EPR signal from such a reduced state is observed, with *g* = [2.18, 2.12, 2.09] and showing well-defined ^51^V (*I* = 7/2) hyperfine splitting, *a*_iso_ = 110 MHz. These findings indicate a different assignment for the electronic structure of the resting state of FeV-cofactor: *S* = 0 (or integer-spin non-Kramers state) with metal-ion valencies, [1V^3+^, 4Fe^3+^, 3Fe^2+^]. Our findings suggest that the V^3+^ does not change valency throughout the catalytic cycle.

## Abstract 2

Active site FeV-cofactor of the V-nitrogenase and the EPR spectrum of the reduced cofactor showing ^51^V-hyperfine coupling.

## Graphical abstract

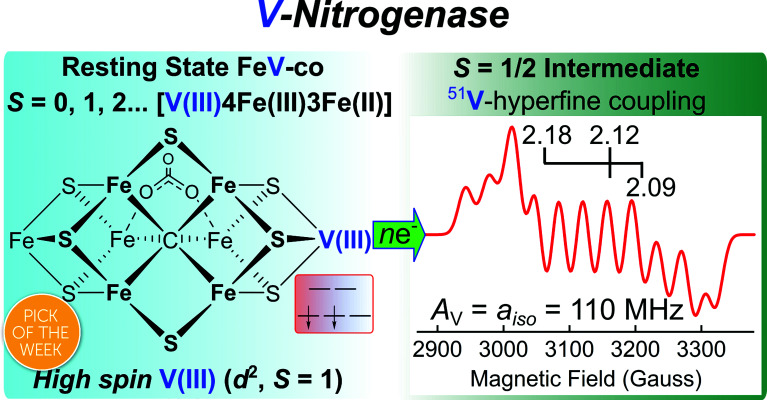

## Introduction

**Fig. 1:**
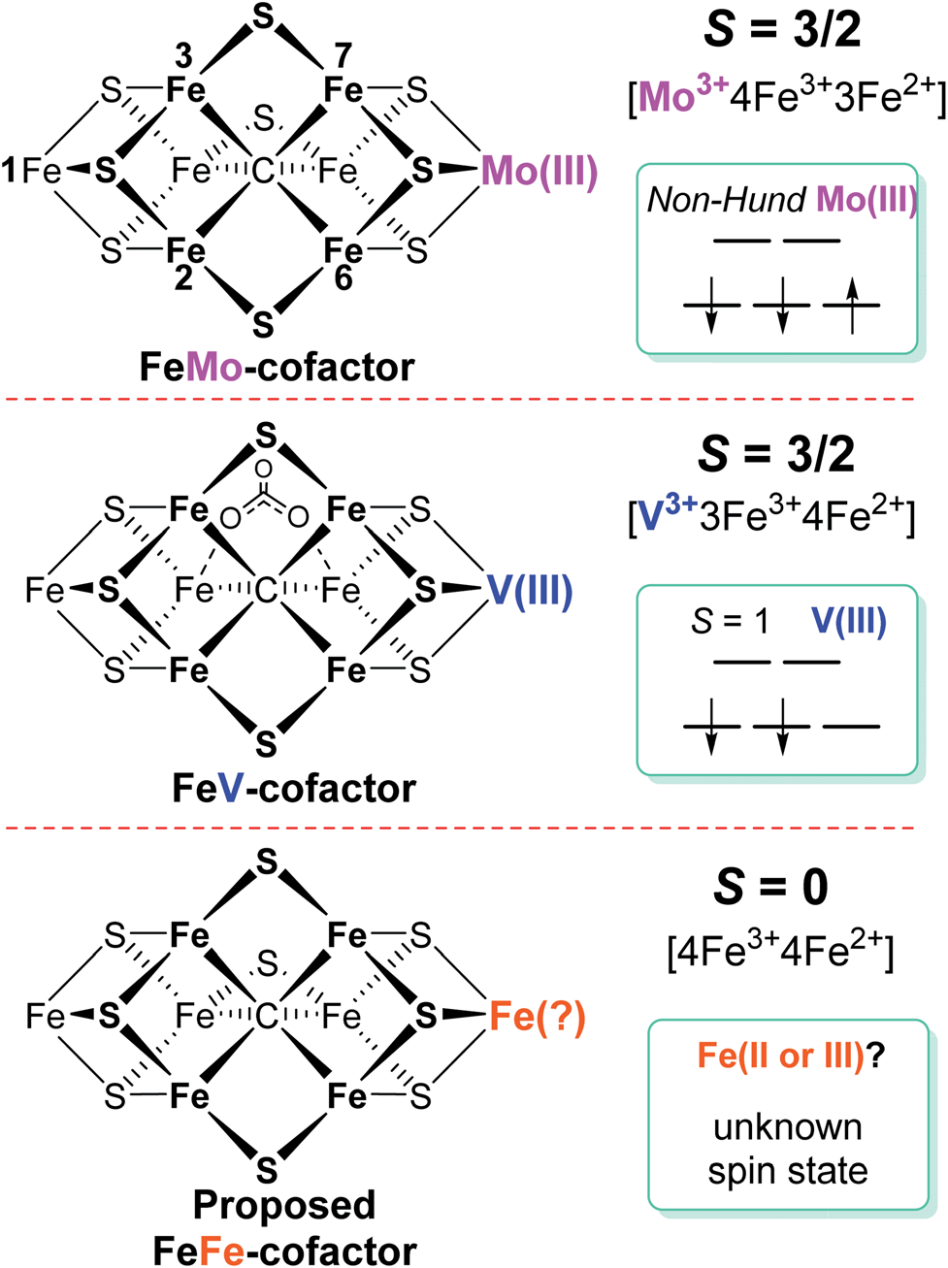
Schematic representation of FeMo-cofactor of Mo-nitrogenase, FeV-cofactor of V-nitrogenase, and proposed FeFe-cofactor in Fe-nitrogenase. The overall spin state of each resting state cofactor, the redox state, and proposed spin state of the Mo, V, and Fe atoms at the same position in the corresponding cofactor are highlighted. The amino acid and *R*-homocitrate ligands are not shown.

Biological nitrogen fixation, the reduction of dinitrogen (N_2_) to ammonia (NH_3_), is catalyzed in diazotrophic bacteria and archaea by the enzyme nitrogenase.^1–3^ Three different nitrogenase isozymes have been described:^4–7^ molybdenum-dependent (encoded by *nif* genes),^8–10^ vanadium-dependent (encoded by *vnf* genes),^5,11–13^ and iron-only (encoded by *anf* genes).^5,14^*Azotobacter vinelandii*, the model organism used in the present work, produces all three different nitrogenase types, although under different physiological conditions, whereas most other nitrogen fixing organisms produce only one or two of the isoenzymes.^15,16^ All three nitrogenase types are binary catalytic systems involving two participating component proteins.^17,18^ One component is responsible for the nucleotide-dependent delivery of electrons to the other component, which provides the active site for substrate binding and reduction. Although genetically encoded by separate genes for the different systems, the electron delivery component for all three systems, products of the *nifH*, *vnfH* and *anfH* genes, is referred to as the “Fe protein”,^13^ whereas the complementary catalytic component for each system are respectively designated the MoFe protein, the VFe protein and the FeFe protein.^4,5,8,11,13^ The MoFe protein is an α_2_β_2_ tetramer, encoded by the *nifD* and *nifK* genes, whereas the VFe protein and FeFe protein are α_2_β_2_δ_2_ hexamers, respectively encoded by the *vnfD*, *vnf*K, *vnfG* and *anfD*, *anf*K, *anfG* genes.^4,5,16,19^ MoFe protein, VFe protein and FeFe protein each contain two types of complex metallo-clusters. One of these is an [8Fe–7S] P-cluster common to all three systems and the other is an active site cofactor, specific to each system and respectively designated FeMo-cofactor, FeV-cofactor, and FeFe-cofactor.^4,5,13^ These designations, as well as the designations of the different systems, reflect the metal compositions of the active site cofactors (Fig. 1). A schematic representation of the V-dependent nitrogenase, the focus of the present work, is shown in Fig. S1,† and the metal-sulfur-carbide core of all three cofactor types and some of their known electronic features is shown in Fig. 1.

**Scheme 1:**
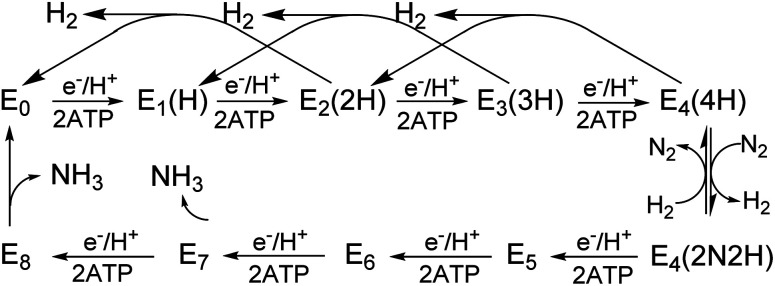
Simplified Lowe-Thorneley kinetic scheme for nitrogen fixation applicable to all three nitrogenase isozymes. The catalytic intermediates of a MoFe (or VFe, or FeFe) protein are denoted E_*n*_, where *n* = 0–8 is the number of [e^−^/H^+^] that have been delivered to the catalytic FeMo-(or FeV-, or FeFe-) cofactor.

All three nitrogenase systems are united by common mechanistic features. Namely, electrons are sequentially transferred through a proposed ‘deficit-spending’ process, with electrons passing from the [4Fe–4S]^1+^ cluster in reduced Fe protein, through the P-cluster, and ultimately accumulated at the active site cofactor. Coupled hydrolysis of a minimum of 2ATP molecules to 2ADP/2Pi occurs for each electron transferred from Fe protein to the corresponding catalytic component.^20,21^ This cycle is repeated until sufficient electrons are accumulated on the active-site cofactor to enable binding and subsequent reduction of substrate. Reduction of different substrates (H^+^, N_2_, C_2_H_2_, CO) varies among the three isozymes.^5,11,22–24^ A recent comparative steady-state kinetic study revealed that all three isozymes follow the same fundamental eight-electron/proton mechanism for N_2_ reduction, with each step involving a cycle of association/dissociation of the two component proteins.^4,20,21,23^ In this scheme, the states of the corresponding catalytic component (MoFe, VFe, or FeFe protein) are denoted by E_*n*_, where *n* represents the number of electrons/protons accumulated on the corresponding active site cofactor (Scheme 1). N_2_ binds at the E_4_ stage after the accumulation of four [e^−^/H^+^], and the reduction of N_2_ is driven by the mechanistically-coupled reductive elimination of two hydrides with the release of H_2_.^23,25^ It was further found that the three isozymes show different ratios of rate constants for this reductive elimination step *versus* the competing hydride protonolysis reaction that only releases H_2_.^4,23^ A determination of the electronic structures and redox properties of the metalloclusters, especially for the corresponding active-site cofactors, is a critical cornerstone in understanding the causes for differences in catalytic properties, as well deciphering common mechanistic features.^4,6^ In the present work, features of FeV-cofactor contained within the VFe protein, in both its resting and turnover states, are explored and compared to known features of FeMo-cofactor and FeFe-cofactor.

Among the three isozymes, Mo-dependent nitrogenase has been the best studied, with details of the mechanism being revealed by a combination of genetic, biochemical, spectroscopic and crystallographic studies.^4,6,8,9,13,20,21,26^ The atomic structure of the active site FeMo-cofactor in the dithionite-reduced (resting state, E_0_) MoFe protein is [7Fe–9S–Mo–C–(*R*)–homocitrate] with an *S* = 3/2 spin state (Fig. 1).^27,28^ Recent X-ray based spectroscopic studies supported the assignment of Mo^3+^ with a non-Hund d^3^ electronic configuration,^29,30^ leading to a preferred charge distribution of the cluster of [Mo^3+^4Fe^3+^3Fe^2+^9S^2−^–C^4−^]^1−^ (Fig. 1).^6,31,32^ Earlier EXAFS and Mӧssbauer studies of resting-state VFe^33–37^ protein and FeFe^14,38^ protein indicated that: (i) both FeV-cofactor and FeFe-cofactor have similar atomic architecture and electronic properties of the 7Fe-subcluster to those of the FeMo-cofactor (Fig. 1);^6^ and (ii) the P-clusters in VFe and FeFe proteins are diamagnetic (*S* = 0), similar to that of their Mo counterpart.^14,37,38^ Recently, a crystal structure of VFe protein has been solved and the modeled structure resembles that for FeMo-cofactor, but with an unusual bidentate four light atomic ligand (assigned as CO_3_^2−^, Fig. 1) replacing one of the belt sulfide (S^2−^) atoms.^13,39^

A Mӧssbauer study suggested that FeFe-cofactor has an even number of ferrous and ferric iron atoms ([4Fe^3+^4Fe^2+^]) with a diamagnetic ground state (*S* = 0), in agreement with the EPR-silence of the resting state FeFe protein.^14,38^ However, there is little consensus on the electronic structure of FeV-cofactor, in part because of contradictory observations of EPR spectra of different preparations of the VFe protein.^5,6,11,13,19^ To date, the FeV-cofactor in the resting state of the enzyme is most typically taken to have *S* = 3/2, and to exhibit a rhombic EPR signal with *g* ≈ 4.3 and 3.8, with the third *g* feature likely hidden under other high field EPR signals.^5,6,13,19,40^ However, the intensity of this signal compared to the *S* = 3/2 signal for resting-state FeMo-cofactor is too low to account for FeV-cofactor in all of the VFe protein present.^41^ Given the suggested *S* = 3/2 spin of the resting state FeV-cofactor, a recent study combining X-ray absorption (XAS) and X-ray emission (XES) spectroscopy and density functional theory (DFT) calculations of resting state MoFe and VFe protein and related synthetic clusters proposed an assignment of V^3+^ with an *S* = 1 d^2^ electronic configuration.^35,42^ Combined with the assignment of FeV-cofactor as EPR-active in the resting state, this implies there must be one more ferrous (Fe^2+^) ion in the Fe_7_ subcluster of FeV-cofactor (Fig. 1) compared to FeMo-cofactor.^6,42,43^

Considering the uncertainties in assigning the origins of the resting-state EPR signal for VFe protein, the electronic structures derived from these spectroscopic and theoretical studies, as summarized in Fig. 1, remained likewise uncertain. Moreover, a proposed assignment of the low spin (*S* = 1/2) EPR signal for resting-state VFe protein to the oxidized P-cluster has been debated.^5,6,13^ The present work is focused on the electronic structures of the FeV-cofactor in both the resting and turnover state of V-nitrogenase. It is revealed that none of the EPR signals previously assigned to FeV-cofactor are consistent with those species representing the dominant active species in the resting state, leading to the conclusion that the resting state FeV-cofactor (E_0_) does not exhibit a half-integer (Kramers) spin state. Rather, freeze-trapping a reduced intermediate formed during turnover reveals an *S* = 1/2 EPR signal showing defined ^51^V hyperfine coupling splitting, leading to the conclusion that this EPR-active partially reduced state of FeV-cofactor (E_1_ or E_3_) has V^3+^ (*S* = 1), and the proposal that this electronic state of vanadium persists throughout the catalytic cycle. These findings lead to a different assignment of iron-ion valencies for FeV-cofactor compared to prior work.

## Experimental

Full experimental details are in the ESI.† This includes *Azotobacter vinelandii* strain construction, cell growth and protein purification, protein activity assays, EPR sample preparation and spectroscopic methods, and ESI† table and figures.

## Results and discussion

### Biochemical characterization of affinity purified VFe protein

**Fig. 2:**
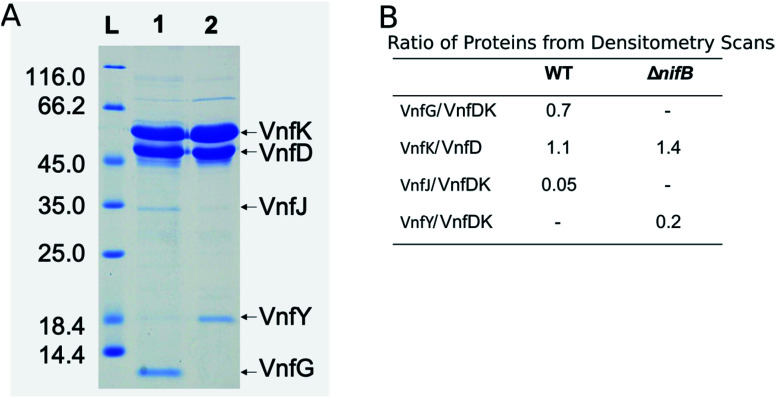
SDS-PAGE of VFe proteins and densitometry data. Panel A. Shown is the molecular weight ladder (lane L) with masses in kDa on the left, VFe^Str^ protein prepared from wild-type (DJ2253) (lane 1), apo-VFe protein from a strain deleted for *nifB* (DJ2256) (lane 2). The positions of the different proteins are shown on the right. Panel B. Reported is the ratio of protein concentrations taken from densitometry scans of Panel A for VFe^Str^ protein α-(VnfD) and β-(VnfK) subunits and copurifying proteins prepared from the wild-type (WT) (DJ2253) and Δ*nifB* strain (DJ2256).

The VFe protein from *A. vinelandii*^40,44,45^ and *Azotobacter chroococcum*^41^ were isolated and studied previously. These earlier studies produced proteins having a range of specific activities and spectroscopic features.^5,6,13^ In the present work, the β-subunit of the VFe protein was genetically modified to include a Strep-tag sequence located near the N-terminal region. Incorporation of a Strep-tag within the β-subunit enabled the rapid and gentle purification of the VFe^Str^ protein, as has been recently demonstrated for several other nitrogen-fixation associated proteins.^10,46,47^ To keep the metal clusters intact from oxygen damage, all proteins were prepared and manipulated under anaerobic conditions and in the presence of dithionite as a reductant.^48^ Panel A in Fig. 2 shows an SDS-PAGE analysis of the VFe^Str^ protein as well as isolated VFe^StrΔ*nifB*^ protein produced by a strain deleted for *nifB*. NifB is required for the formation of NifB-co, an [8Fe–9S–C] precursor required for formation of all nitrogenase active site cofactor types.^5,10^ Inspection of the VFe^Str^ protein profile (Fig. 2, lane 1) reveals that it co-purifies with a minor sub-stoichiometric amount of VnfJ. The gene encoding VnfJ, a designation assigned in the present work, is located immediately downstream of the *vnfK* gene (encoding the β-subunit of the VFe protein) and precedes *vnfY*. The function of VnfJ is not known, but its co-purification in very small amounts with the VFe^Str^ protein indicates it is likely to be involved in some aspect of VFe protein maturation and that a small amount of intermediate assembly species is captured by the affinity purification procedure. Based on densitometry, the approximate subunit composition of the isolated VFe^Str^ protein is α_2_β_2_δ_2_, which is in line with the organization and apparent translational coupling of the corresponding genes,^49^ the composition evident from the crystal structure,^13,39,50^ as well as the composition reported by other investigators,^40,41,51^ with the exception of Lee *et al.*,^45^ who claim an α_2_β_2_δ_4_ composition.

Affinity purification of VFe-protein produced by a *nifB*-deletion strain results in loss of the δ-subunit, encoded by *vnfG*, and sub-stochiometric co-purification with VnfY. VnfY has a similar primary structure when compared to NifY/NafY from the Mo-dependent system. Similar to the situation found here, NifY/NafY, which are proposed to be FeMo-cofactor trafficking proteins,^10^ co-purifies with MoFe protein produced by the *nifB*-deletion strain.^46,52^ It, therefore, appears that VnfY has a role related to FeV-cofactor trafficking/insertion during maturation of the VFe protein, which is also consistent with prior biochemical phenotype of a strain deleted for *vnfY*.^53^ Another feature of VFe-protein produced by the *nifB*-deletion strain is that it apparently accumulates as a mixture of α_2_β_2_ and α_1_β_2_ species based on the differential intensity of bands corresponding to the α- and β-subunits shown in lane 2 of Fig. 2, which, again, is consistent with previous reports.^5,12,44,54^

A comparison of the specific activities of the VFe proteins used in the present work for reduction of the physiological substrates, N_2_ and protons, is shown in Table 1. The VFe^Str^ protein shows specific activities for N_2_ and proton reduction consistent with the highest reported values from prior studies,^5^ whereas, the VFe^StrΔ*nifB*^ protein, which does not contain FeV-cofactor, has no N_2_ or proton reduction capacity. For reasons described in a following section, VFe protein produced by a strain having *nifE* deleted (VFe^StrΔ*nifE*^) was also isolated. NifE is specifically required for formation of the active site FeMo-cofactor associated with the Mo-dependent nitrogenase. The VFe^StrΔ*nifE*^ protein sample exhibited lower activities relative to the VFe^Str^ protein for reduction of N_2_ and protons, but well within the variability we have routinely observed and others have reported^5,40^ for VFe protein preparations.

**Table 1:** Specific activities of VFe proteins at pH 7.3^a^

VFe protein	Substrates		
	Protons (1 atm Ar)	N_2_ (1 atm) and protons	
	nmol of H_2_ min^−1^mg^−1^	nmol of H_2_ min^−1^mg^−1^	nmol of NH_3_ min^−1^mg^−1^
VFe^Str^	1980 ± 50	960 ± 25	310 ± 7
VFe^StrΔ*nifB*^	ND^b^	ND	ND
VFe^StrΔ*nifE*^	1110 ± 1	690 ± 3	160 ± 5
*In vitro* incubated VFe^StrΔ*nifE*^	1240 ± 27	700 ± 8	200 ± 9

a All assays were performed at 30 °C for 8 min at a molar ratio of VFe protein to VnfH of 1 : 40 (1 : 30 for VFe^StrΔ*nifB*^), and the specific activities are expressed as nmol of product per min per mg of VFe protein as an average with standard deviation.

b ND = not detected.

### Analysis of the *g*-2 region *S* = 1/2 EPR signal associated with isolated VFe protein

**Fig. 3:**
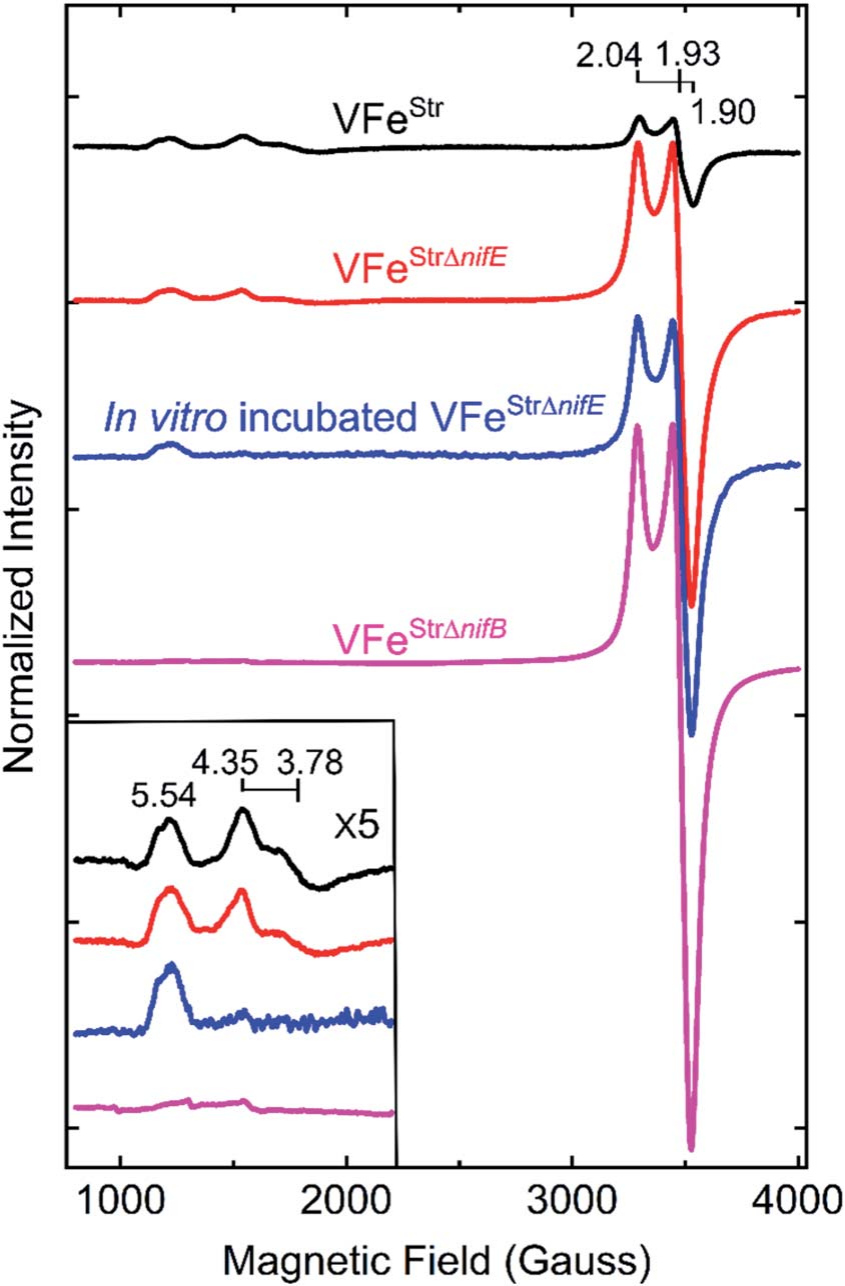
X-band EPR spectra of resting state strep-tagged VFe proteins. The EPR spectra for 50 μM of VFe^Str^ protein (VFe^Str^, black trace), for 52 μM of VFe^Str^ protein from a *nifE*-deleted genetic background (VFe^StrΔ*nifE*^, red trace), for 48 μM of *in vitro* incubated Strep-tagged VFe protein from an *nifE*-deleted genetic background (*in vitro* incubated VFe^StrΔ*nifE*^, blue trace), and for 50 μM VFe^Str^ protein from an *nifB*-deleted genetic background (VFe^StrΔ*nifB*^, magenta trace) are presented. The details for creation of the different strains with different genotypes and the *in vitro* incubation experiment are found in the ESI.† All samples were made in a 100 mM MOPS buffer, pH 7.3, with *ca.* 20 mM sodium dithionite and 150 mM NaCl. Inset shows an expansion of the low field region. EPR conditions: temperature, 12 K; microwave frequency, 9.38 GHz; microwave power, 20 mW; modulation amplitude, 8.14 G; time constant 20.48 ms. Each trace is the sum of five scans.

The X-band EPR spectrum for the resting state, dithionite reduced VFe^Str^ is shown in Fig. 3. This protein shows an *S* = 1/2 EPR signal in the *g*-2 region with *g* = [2.04, 1.93, 1.90]. Spin quantification of the *S* = 1/2 signal indicated ∼0.3 electron spins per VFe^Str^ protein, consistent with the previous reports.^5,19,45^ Although the origin and catalytic relevance of the *S* = 1/2 EPR signal associated with P-clusters is not the focus of the present work, the highly variable intensity of this signal apparent in different VFe protein preparations, as shown in Fig. 3, merits some discussion. As evident from SDS-PAGE analysis of purified VFe protein samples shown in Fig. 2, there is not a strict equivalence in the relative α- and β-subunit composition in preparations of VFe protein described here and elsewhere.^5^ Namely, the β-subunit often appears to be present in excess of the α-subunit, which is consistent with the presence of both α_1_β_2_ and α_2_β_2_ species in such samples. In our hands, the intensity of the *S* = 1/2 signal is roughly correlated with an increase in sample heterogeneity. This observation is similar to reports in the pioneering work from the Hales laboratory. In those studies,^44,51^ it was shown that an α_1_β_2_ VFe protein species could be isolated and that the “spare” β-subunit within that complex appears to contain a [4Fe–4S] P-cluster “fragment” having features very similar to the *S* = 1/2 signal reported here. It is also possible that the *S* = 1/2 signal is associated with P-cluster precursors similar to those found in immature MoFe protein produced by a *nifH*-deficient strain.^46,47^ Although the true origin of the variable *S* = 1/2 EPR signature associated with isolated resting state VFe protein, and its possible relevance to catalysis or P-cluster assembly, remains to be resolved, there is compelling evidence and agreement that it is not associated with the active site FeV-cofactor because it persists in VFe protein prepared from a *nif*B-deficient strain that cannot produce FeV-cofactor (Fig. 3).^5,54^

### Analysis of the low field *S* = 3/2 EPR signals associated with VFe protein

The EPR spectrum of the resting-state VFe^Str^ protein shows weak signals at lower field (high *g* values) that correspond to previously recognized *S* = 3/2 spin states, with *g* features at 5.54, 4.35, and 3.78 (Fig. 3). These EPR signals were initially proposed to be a mixture of *S* = 3/2 species, presumably reflecting different protein environments of the active site, FeV-cofactor.^11,19,41^ In a recent study, similar *S* = 3/2 signals have been clearly distinguished as two species according to their different temperature dependence behavior and different redox response to indigo disulfonate (IDS).^45^ The *S* = 3/2 signals observed here show very low intensities, in agreement with the previous estimation that these signals correspond to less than 10% of the intensity of the similar signals of the *S* = 3/2 FeMo-cofactor signal present in MoFe protein.^19,41^

The low intensity of the *S* = 3/2 signals raises doubts about their assignment to resting state FeV-cofactor. We, therefore, explored the possibility that these signals could arise from mis-incorporation of FeMo-cofactor into the VFe protein, given the similarities of *g* values to those arising from FeMo-cofactor in MoFe protein. To test this possibility, a VFe protein was isolated from *A. vinelandii* cells having the *nifE* gene deleted (VFe^StrΔ*nifE*^). Deletion of *nifE* disables formation of FeMo-cofactor, removing any possibility of its misincorporation into VFe protein. The VFe^StrΔ*nifE*^ protein exhibits essentially the same EPR features in the *S* = 3/2 and *S* = 1/2 regions as VFe^Str^ protein, although having a more prominent *S* = 1/2 signal compared to the *S* = 3/2 signal (Fig. 3). This observation rules out the possibility that the *S* = 3/2 signal might originate from FeMo-cofactor.

Expressing the VFe^Str^ protein in a background having *nifB* deleted results in formation of VFe^StrΔ*nifB*^ protein that does not contain any FeV-cofactor. The EPR spectrum of this protein shows the persistence of the *S* = 1/2 signal, but loss of the *S* = 3/2, *g* 5.54 and *g* 4.35 and 3.78 signals (Fig. 3). As already described, this finding is consistent with assignment of the *S* = 1/2 signal to P-cluster, or a species associated with either P-cluster precursor or damaged P-cluster. Disappearance of the *S* = 3/2 features in isolated VFe^StrΔ*nifB*^ protein indicates that the low field EPR signals must originate either from FeV-cofactor or one of its intermediate assembly species.

To test whether the *S* = 3/2 signals might be associated with a VFe^Str^ protein species that contains an immature form of FeV-cofactor, crude extract prepared from the strain producing VFe^StrΔ*nifE*^ was supplemented with V and α-ketoglutarate and incubated for 4 h under turnover conditions prior to isolation of VFe^StrΔ*nifE*^. Extracts prepared from a strain deleted for nifE was used for this experiment to ensure there was no possibility for adventitious incorporation of FeMo-cofactor into VFe protein during the incubation. Such incubation resulted in only a slight increment in specific activity of the isolated protein (Table 1), but also a loss in the *g* 4.35 and 3.78 EPR signals (Fig. 3). The *g* 4.35 and 3.78 signals are thus not correlated with active protein, evidence against the assignment of these signals as the resting state of the active form of FeV-cofactor.^5^ Even though the chemistry behind the loss in the *g* 4.35 and 3.78 EPR signals is not yet understood, the result suggests these signals are not associated with the active form of FeV-cofactor.

**Fig. 4:**
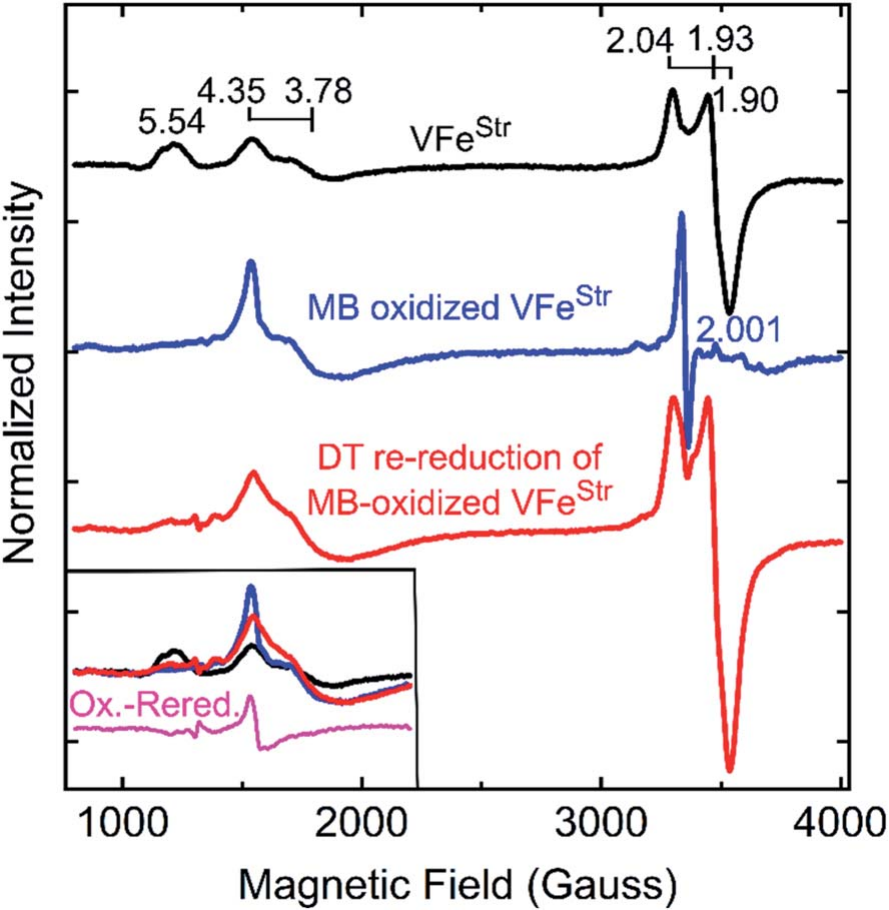
X-band EPR spectra of oxidized VFe proteins. Shown is the resting state VFe^Str^ in MOPS buffer with 20 mM DT (black trace), methylene blue (MB)-oxidized VFe^Str^ after 15 min incubation at room temperature in MOPS buffer without DT (blue trace), and 20 mM DT re-reduced VFe^Str^ after being oxidized by MB for 15 min (red trace). The MOPS buffer was 100 mM, pH 7.3, with *ca.* 150 mM NaCl. The final VFe^Str^ concentration was 50 μM in all samples. The inset in the left bottom corner presents an overlay of the low field region of the spectra from three spectra and the spectra difference (magenta trace) between the MB-oxidized sample and DT re-reduced sample after MB oxidation. EPR conditions are the same as those in Fig. 3.

The feature at *g* = 5.54 might originate from the overlap of the two signals with *g* ≈ 5.7 and 5.4 arising from the ground and excited state of an inverted *S* = 3/2 system.^40^ The assignment of this signal was probed by redox cycling of the VFe protein. Methylene blue (MB) is able to oxidize the resting state FeMo-cofactor (M^N^) and P-cluster (P^N^) in MoFe protein to a diamagnetic EPR silent M^ox^ state and a paramagnetic P^ox^ state, respectively.^55^ After a 15 min treatment of VFe^Str^ with MB, the high spin *S* = 3/2 (*g* = 5.54) and low spin *S* = 1/2 (*g* = 2.04, 1.93, and 1.90) signals disappear in the EPR spectrum (Fig. 4). However, the *S* = 3/2 (*g* 4.35) signal remained after oxidation, accompanied by the appearance of an adventitious *S* = 5/2 Fe(iii) species with a signal at *g* ≈ 4.3 (Fig. 4). Re-reduction of the oxidized VFe^Str^ protein by 20 mM dithionite resulted in an unchanged *S* = 3/2 (*g* = 4.35) signal, and the recovery of the *S* = 1/2 signal, but not the *S* = 3/2 (*g* = 5.54) signal (Fig. 4). A broad but weak feature, different from the line-shape of the aforementioned *S* = 3/2 (*g* = 5.54) signal, appears in the EPR spectrum after the re-reduction by dithionite (Fig. 4). This broad feature ranges from *g* ∼ 5.8 to *g* ∼ 5.1. Careful examination of the line-shape revealed that this feature is quite similar to that for the *S* = 3/2 spin state of the [4Fe4S]^1+^ cluster Fe protein produced by the Mo-dependent system, which can be reversibly converted to the *S* = 1/2 spin state of the [4Fe4S]^1+^ cluster of Fe protein.^6,56^ The origin of this *S* = 3/2 signal is not yet clear. Because the appearance of this signal (*g* ∼ 5.8 to *g* ∼ 5.1) accompanies the recovery of the *S* = 1/2 signal after the dithionite re-reduction of MB-oxidized VFe^Str^, it is reasonable to propose that this new signal originates from the high spin form of the metal cluster displaying the *S* = 1/2 signal (*g* = 2.04, 1.93, and 1.90). VFe^Str^ protein that was MB oxidized and then reduced by dithionite maintained about half of substrate reducing activities of that for non-oxidized VFe^Str^ protein (see Table S1†) yet it lost the *g =* 5.54 EPR signal. Thus, it can be concluded that this signal does not arise from the catalytically active FeV-cofactor.

The two *S* = 3/2 signals, from the *g* = 5.54 and *g* = 4.35 and 3.78 species, long seen in VFe preparations and ascribed to resting-state FeV-cofactor can be ruled out as arising from catalytically relevant FeV-cofactor based on the results presented here. Even though contradictory results for these signals have been reported, the intensity of the *S* = 3/2, *g* = 4.35 and 3.78 signals were still used as an indicator to distinguish the dithionite-reduced, ‘resting’ state from a ‘turnover’ state in the crystallographic study of VFe protein.^13,50^

### Spin state and valencies of resting-state FeV-cofactor

The proposal of the dithionite-reduced resting state of FeV-cofactor as an *S* = 3/2 EPR-active system resulted in an assignment of the valency of the resting FeV-cofactor as [V^3+^3Fe^3+^4Fe^2+^] with one more iron site in its ferrous state than that of resting FeMo-cofactor ([Mo^3+^4Fe^3+^3Fe^2+^]) based on XAS and DFT studies.^6,42,43^ However, there are several important observations that contradict assignment of an *S* = 3/2 state to the resting FeV-cofactor: (i) unlike the quantitative FeMo-cofactor EPR signal of MoFe protein, the *g* = 4.3, *S* = 3/2 signal of VFe protein has always been observed with low, and varying intensity,^5,13,19,41,45,50,51^ being absent in some isolations;^12,44,57,58^ and (ii) the different redox responses of the two *S* = 3/2 spin systems seen with VFe protein toward the oxidation by IDS^45^ and methylene blue described here demonstrate that the two *S* = 3/2 signals do not originate from the catalytically relevant FeV-cofactor. Thus, as described above (Table 1, Fig. 3 and 4), it is clear none of the *S* = 3/2 species seen in preparations of VFe protein are necessarily relevant to catalytically functional FeV-cofactor. In aggregate, the results reported here indicate that the two *S* = 3/2 species observed in dithionite-reduced VFe proteins originate either from incomplete/immature FeV-cofactor or from some adventitious EPR active species. As none of the EPR signals evident in dithionite-reduced VFe protein originate from functional FeV-cofactor, we are led to conclude that the dithionite-reduced FeV-cofactor in resting-state VFe protein most likely diamagnetic, but possibly in an integer-spin (non-Kramers) state.

### Reduced states of the VFe protein

**Fig. 5:**
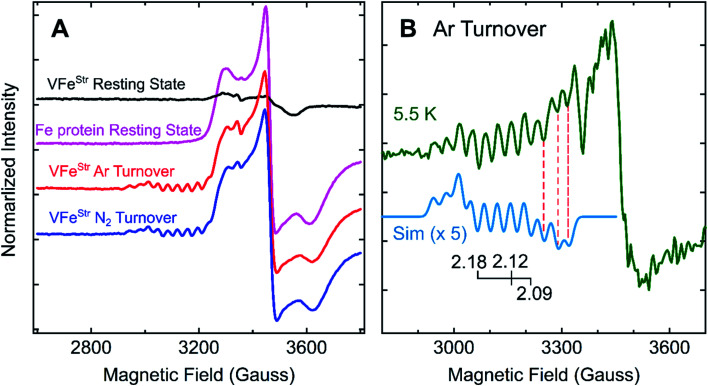
*g*-2 region X-band EPR spectra of V-nitrogenase proteins. (A) 12 K spectra of resting states (black trace for VFe^Str^ and magenta trace for Fe protein) and Fe protein-VFe^Str^ freeze-trapped during turnover under Ar (red trace) and N_2_ (blue trace) (B) 5.5 K (green trace) spectra of Ar-turnover Fe protein–VFe^Str^ along with simulation of turnover intermediate (cyan trace) with ^51^V hyperfine coupling, *a*_iso_ = 110 MHz, *g* = [2.18, 2.12, 2.09], and isotropic EPR linewidth of 75 MHz. Samples: all samples contain 5 μM of VFe^Str^ protein except the Fe protein resting state sample, and 40 μM of Fe protein except the VFe^Str^ resting state sample. All samples made in buffer with 200 mM MOPS at pH 7.3 and an ATP-regeneration system (20 mM ATP, 20 mM MgCl_2_, 1 mg mL^−1^ BSA, and 0.4 mg mL^−1^ creatine phosphokinase) with a final dithionite concentration at ∼20 mM. EPR conditions: as in Fig. 3 except each spectrum is the sum of 10 scans.

Freeze-trapping nitrogenase under high-flux turnover conditions with Fe protein and ATP results in the capture of reduced states of the active site cofactor (E_*n*_, *n* > 0; Scheme 1).^4,9,26^ Spectroscopic studies of reaction intermediates freeze-trapped during turnover of Mo- dependent nitrogenase have revealed all EPR-active E_*n*_ states (*n* = even) except E_6_.^4^ To date, no such turnover intermediates have been trapped and characterized for V-nitrogenase.^5,6^ The 12 K EPR spectra of 5 μM VFe^Str^ protein freeze-trapped during turnover under Ar or N_2_ show not only a strong signal from the [4Fe–4S]^1+^ cluster of the Fe protein, but also, to the low-field side of that signal, there appears a portion of a partially overlapping *S* = 1/2 signal with much smaller amplitude exhibiting multiple well-defined ^51^V (*I* = 7/2) hyperfine-splittings (Fig. 5A and S2†). It is noteworthy that the newly observed signal with ^51^V hyperfine splitting is the same whether observed during Ar or N_2_ turnover, and the intensity of this signal increases with increasing Fe protein concentration (or increasing electron flux) as depicted in Fig. S3.† The intensity of the signal from the turnover intermediate does not significantly change until the temperature is increased to 16 K (Fig. S4†) and enhanced spin-lattice relaxation occurs.

Given the observation of ^51^V-hyperfine splitting, it can be concluded that the newly observed *S* = 1/2 signal arises from FeV-cofactor. The increase in intensity with increasing electron flux indicates that the signal is from a reduced intermediate. Confirmation that this signal is indeed associated with a catalytic E_*n*_ intermediate (Scheme 1) was provided by using EDTA to quench electron delivery from the Fe protein, which resulted in decay of the turnover dependent EPR signal (Fig. S5†). Confidence that EDTA quenching does not affect cluster composition is provided by the observation the EPR signature of the most sensitive cluster in the system, the Fe protein [4Fe–4S] cluster, is unaffected by the quenching procedure. An earlier report showed no appearance of a new *S* = 1/2 signal under turnover, much less one that exhibited ^51^V hyperfine structure.^45^ In that study, the turnover experiment was performed using low-flux conditions,^45^ whereas the turnover experiment reported here was performed using high-flux conditions. Although low-flux turnover experiments with MoFe protein have proven useful in the capture of the E_1_ state,^59,60^ they were not able to populate more highly reduced states of the enzyme.^4,9^

Careful inspection of the EPR spectrum of the intermediate shows the two ^51^V hyperfine lines at lowest field have the ‘absorption’ shape characteristic of components in a ^51^V (*I* = 7/2) octet associated with the *g*_1_ feature for an *S* = 1/2 center having a rhombic g-tensor, while the remaining ^51^V hyperfine lines have the derivative shape of the octet from the *g*_2_ feature. This pattern fixes both [*g*_1_, *A*_1_] and [*g*_2_, *A*_2_], but the strong Fe protein signal at 12 K precludes any insights into the values of [*g*_3_, *A*_3_]. However, as the temperature is lowered, the Fe protein signal saturates and decreases in amplitude, while that of the intermediate does not (Fig. 5B and S4†). At the base temperature of 5.5–6 K, at fields directly above the *g*_2_ octet, two additional negative going ^51^V hyperfine lines are observed, the shape expected for *g*_3_ features, with no indication of additional ^51^V lines to still higher field. With this guidance, the observed intermediate spectra can be simulated quite well with g-tensor, *g* = [2.18, 2.12, 2.09], and an isotropic ^51^V hyperfine tensor, *A* = *a*_iso_ = 110 MHz, Fig. 5B and S6.† Together, the absence of this *S* = 1/2 signal with ^51^V hyperfine splitting in the EPR spectrum of resting state VFe^Str^ and its appearance under turnover conditions indicate that this signal arises from FeV-cofactor of a turnover trapped state.

To estimate the degree of accumulation of this newly identified EPR-active intermediate, it is found that the simulated sum of the hyperfine-split signal (HSS) and Fe protein signal is achieved by adding them in the intensity ratio, HSS/Fe protein ∼1/17 (Fig. S6†). Given that the turnover conditions include high reductant concentration (20 mM dithionite) and high Fe protein concentration, it is reasonable to infer that Fe protein is overwhelmingly present in its EPR-active reduced state, and this is confirmed by direct integration of the signal to 0.48 spin per Fe protein. In the case FeV-cofactor, the relative contributions to the simulation imply that roughly 10% of that present (total, 5 μM VFe protein, 10 μM FeV-cofactor) has been trapped as the newly identified EPR active intermediate.

### E_*n*_ state assignment of the newly observed intermediate

Because the intermediate is generated during turnover under an Ar or N_2_ atmosphere, this intermediate must be E_*n*_ where *n* ≤ 4, as E_5–8_ states only exist in the presence of N_2_ (Scheme 1). Given that the resting E_0_ state of VFe-protein is diamagnetic or in a non-Kramers state, then states having an even number of added electrons, E_4_ and E_2_, should also be EPR silent. As a result, the *S* = 1/2 intermediate trapped here would be E_1_(1H) or E_3_(3H), namely reduced from resting state by one or three electrons, and hence is denoted the E_1,3_(1,3H) state.

### Vanadium valence state in E_1,3_(1,3H)

The presence of the large ^51^V hyperfine coupling seen in Fig. 5 for a vanadium ion incorporated into a paramagnetic FeV-cofactor requires that this ion itself be in a paramagnetic valence state with a large hyperfine coupling. There are only two plausible paramagnetic states for such a V ion: V^3+^ (d^2^, *S* = 1) or V^4+^ (d^1^, *S* = 1/2). Comparison of the observed ^51^V hyperfine coupling in the turnover intermediate with those of reference compounds is next shown to confirm the signal indeed is associated with FeV-cofactor and to identify the valency of vanadium.

This effort begins with the recognition that the experimentally observed hyperfine coupling tensor for the nucleus of metal-ion site i, *A*i, within the multinuclear spin-coupled FeV-cofactor cluster is proportional to the intrinsic hyperfine coupling tensor for the uncoupled (isolated) metal ion, *A*^un^_i_, as scaled by the projection of the metal ion's local spin onto the total cluster spin. This dimensionless constant, denoted the vector-coupling coefficient, *K*_i_, is subject to a normalization condition on the sum over the *K*_i_ for the coupled metal ions;

1 *A*_i_ = *K*_i_*A*^un^_i_ ; Σ*K*_i_ = 1

According to eqn (1), spin coupling within a cluster alters the *magnitude* of the nuclear hyperfine interaction, not its ‘*symmetry*’: isotropic; axial; rhombic. The V^3+^ (d^2^, *S* = 1) valence state, which is relatively rare, has been found to exhibit an isotropic coupling (*a*^un^_iso_(iii) ≅ 300 MHz).^61^ The V^4+^ (d^1^, *S* = 1/2) state is quite common, and is well-known to show an extremely anisotropic (roughly axial) hyperfine tensor (component values that range around median values of, *A*_*‖*_*∼*500 MHz, *A*_⊥_ ∼200 MHz, *a*^un^_iso_ ∼300 MHz).^62^ The finding that ^51^V hyperfine coupling of E_1,3_(1,3H) is fully isotropic then identifies the vanadium of E_1,3_(1,3H) as V^3+^ (d^2^, *S* = 1).^35,42,43^

The isotropic ^51^V hyperfine coupling observed in the E_1,3_(1,3H) intermediate, *a*_iso_ = 110 MHz, is much smaller than that of an isolated V^3+^, *a*^un^_V_ ∼300 MHz, and furthermore, the intermediate signal is from an *S* = 1/2 (Kramers) center, not that of an isolated V^3+^ (non-Kramers) *S* = 1 center. These observations together confirm that the observed signal comes not from an isolated V^3+^ (d^2^, *S* = 1) complex produced by cofactor degradation, but from a spin-coupled multi-metallic cluster, a state of spin-coupled FeV-cofactor itself, and thus indeed from a VFe intermediate. Taking eqn (1) and using *A*^un^_V_ = *a*^un^_iso_ ∼300 MHz for an isolated V^3+^ (d^2^, *S* = 1), then *a*_iso_ = 110 MHz for the intermediate yields a vector-coupling coefficient for the V^3+^ of E_1,3_(1,3H): *|K*_V(iii)_|∼0.3. ENDOR measurements of the sign of the ^51^V hyperfine coupling will establish the sign of this coefficient.

As an instructive exercise designed to illuminate the spin properties of this intermediate, we examine two alternative limiting models for spin coupling within a FeV-cofactor with cluster spin, *S* = 1/2 and containing V^3+^ (*S* = 1): simple antiferromagnetic coupling between a V^3+^ (*S* = 1) and a Fe_7_ subcluster would yield the observed *S* = 1/2 cluster spin if the subcluster had a net spin of either *S*(Fe_7_) = 1/2 or 3/2. It is straightforward to show that such coupling to a subcluster-spin *S*(Fe_7_) = 1/2 would give a vanadium spin-projection coefficient greater than unity, *K*_V_ = 4/3, and thus a V^3+^ hyperfine coupling greater than that of an uncoupled ion, contrary to observation. In contrast, antiferromagnetic coupling to a subcluster spin *S*(Fe_7_) = 3/2 would give |*K*_V_| = 2/3 < 1 (actually, *K*_V_ < 0), and thus a V^3+^ hyperfine coupling less than that of an uncoupled ion, a result that qualitatively, even though not quantitatively, reflects experiment. This exercise shows how the observed hyperfine coupling can be used to gain a qualitative understanding of how the V^3+^ (*S* = 1) in E_1,3_(1,3H) is anti-ferromagnetically coupled to an Fe_7_ sub-cluster of FeV-cofactor with an overall spin *S* = 3/2. A full treatment of spin coupling within FeV-cofactor, which is not at present accessible, would be needed to precisely discuss all the metal-ion hyperfine couplings and the E_1,3_(1,3H) g-tensor. Such a more complex scheme would explicitly incorporate contributions to its magnetic properties from zero-field splittings on both Fe and V.

**Fig. 6:**
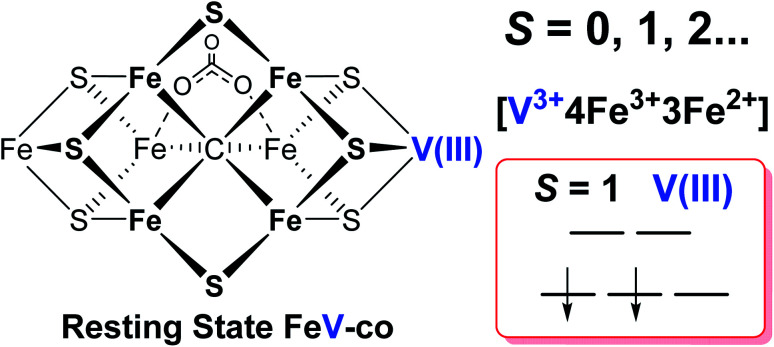
Schematic representation and electronic assignment of the dithionite-reduced resting state (E_0_) FeV-cofactor. The proposed overall electron spin state, metal valences, and d-orbital electronic configuration of high spin V(iii) (d^2^, *S* = 1) are highlighted on the right.

It has been shown that E_0_ of FeMo-cofactor contains a d^3^ Mo^3+^ (Fig. 1),^29,30,63^ which is odd-electron, that in forming the MoFe E_1_(H) the molybdenum remains Mo^3+^,^60,64^ and further, it has been proposed that this valency persists throughout the catalytic cycle.^65^ Turning to FeV-cofactor, it is shown here the odd-electron (*S* = 1/2) E_1,3_(1,3H) state contains an even-electron V^3+^ (*S* = 1). It is proposed, in part by analogy to MoFe protein, that the V^3+^ valency likewise persists throughout the catalytic cycle, in which case the difference in spin states and EPR behavior observed for the E_*n*_ states of FeMo-cofactor and FeV-cofactor arise because the two cofactors exhibit the same, unchanging trivalent state for the heterometal throughout the cycle. Simply put, it is suggested that the difference between the overall cluster spin states for the E_*n*_ states of FeMo-cofactor and FeV-cofactor arises merely because Mo^3+^ is an odd-electron Kramers ion (half-integer spin), whereas V^3+^ is an even-electron, non-Kramers (integer-spin) ion, while the overall valencies of the Fe_7_ sub-cluster are the same in the corresponding E_*n*_ states of the two cofactors. Taken together, the results in this work suggest that the dithionite-reduced resting state (E_0_) FeV-cofactor is diamagnetic (*S* = 0) or paramagnetic with integer-spin (*S* = 1, 2…), with a high spin V^3+^ (d^2^, *S* = 1) ion, and four ferric (Fe^3+^) and three ferrous (Fe^2+^) ions in the Fe_7_ sub-cluster (Fig. 6).^31,32^ These findings are consistent with the electronic similarities suggested by iron-selective Mӧssbauer study of V-dependent nitrogenase,^37^ but contradict the suggestion of electronic structure differences of the Fe_7_ subclusters in FeMo-cofactor and FeV-cofactor based on XAS and DFT studies.^6,42,43^

## Conclusions

A combination of genetic, biochemical, and biophysical studies on V-dependent nitrogenase has revealed that the *S* = 3/2 and *S* = 1/2 signals long observed in the EPR spectra of dithionite-reduced, resting-state VFe protein are probably not associated with a functional FeV-cofactor. In contrast to the (*S* = 3/2) resting state FeMo-cofactor of Mo-nitrogenase, FeV-cofactor in the dithionite-reduced VFe protein can now be described as likely diamagnetic (*S* = 0), similar to that of the FeFe-cofactor of Fe-nitrogenase. The active P-cluster in VFe protein is likely to be diamagnetic, the same as are those in Mo- and Fe-nitrogenase,^6,8^ in agreement with Mӧssbauer studies.^37^

Under turnover conditions, an *S* = 1/2 spin state intermediate (*g* = [2.18, 2.12, 2.09]) that forms prior to N_2_ binding (Scheme 1) has been trapped and is assigned to a state reduced by an odd number of electron: E_*n*_, *n* = 1 or 3. The well-defined ^51^V hyperfine coupling seen for this intermediate show it to have a V^3+^ (d^2^, *S* = 1) valence. It further shows that the V(iii) ion is antiferromagnetically spin-coupled to Fe ions of the Fe_7_ subcluster of FeV-cofactor, with the Fe ions themselves instructively discussed as being coupled into what is in effect a spin of *S* = 3/2. Given that the resting state of FeMo-cofactor is persuasively assigned valences of [Mo^3+^, 4Fe^3+^, 3Fe^2+^], the most plausible assumption is that the resting-state FeV-cofactor instead has metal-ion valences: [V^3+^, 4Fe^3+^, 3Fe^2+^].

## Author contributions

Z.-Y. Y. proposed the research direction. E. J.-V., J. S. M. D. C., Z.-Y. Y., and H. K. and were responsible for bacterial strain constructions and protein purification. Z.-Y. Y. and H. K. were responsible for biochemical and EPR studies. H. Y. and D. A. L. did EPR spectral simulations. Z.-Y. Y., D. A. L., B. M. H., D. R. D., and L. C. S. were responsible for the original manuscript draft and revisions of the manuscript with input from E. J.-V., H. K., H. Y., and J. S. M. D. C. All authors have approved the revisions and submission of the manuscript.

## Conflicts of interest

There are no conflicts to declare.

## Supplementary Material

SC-012-D0SC06561G-s001
