# Supplementary material for: The electronic structure of FeV-cofactor in vanadium-dependent nitrogenase
Source: Chem Sci. 2021 Mar 29;12(20):6913–22. doi: 10.1039/d0sc06561g (PMC8153082; doi:10.1039/d0sc06561g)
Supplement: SC-012-D0SC06561G-s001 [file SC-012-D0SC06561G-s001.pdf]

Electronic Supplementary Material (ESI) for Chemical Science.
This journal is © The Royal Society of Chemistry 2021

Supporting Information

The Electronic Structure of FeV-cofactor in Vanadium-Dependent Nitrogenase

Zhi-Yong Yang,[*a] Emilio Jimenez-Vicente,[b] Hayden Kallas,[a] Dmitriy A. Lukoyanov,[c] Hao Yang,[c] Julia S. Martin del Campo,[b] Dennis R. Dean,[*b] Brian M. Hoffman,[*c] and Lance C. Seefeldt[*a]

Author affiliations:
[a]Department of Chemistry and Biochemistry, Utah State University, Logan UT 84322;
[b]Department of Biochemistry, Virginia Tech, Blacksburg, VA 24061;
[c]Department of Chemistry, Northwestern University, Evanston, IL 60208.

Materials and methods

Reagents and general procedures. Unless otherwise stated, all reagents were purchased from Sigma-Aldrich (St. Louis, MO) and used without further purification. All gases (hydrogen, argon, and dinitrogen) were purchased from Air Liquide America Specialty Gases LLC (Plumsteadville, PA). The argon and dinitrogen gases were passed through an activated copper catalyst to remove trace dioxygen contamination prior to use. All proteins, solutions, and buffers were handled anaerobically under an inert atmosphere (argon or dinitrogen) on a Schlenk vacuum line. The transfer of gases and solutions was done anaerobically with Hamilton gas-tight syringes.

***Azotobacter vinelandii* strain construction**. Strain DJ33,[1] which carries an in-frame *nifD-nifK* deletion, was transformed with genomic DNA isolated from the W-tolerant strain CA11.6[2,3] and transformants selected for growth in the presence of 1mM W without addition of a fixed nitrogen source nor a Mo supplement in order to transfer the W-tolerant phenotype to DJ33. W-tolerance relieves the capacity for trace levels of Mo in the growth medium to suppress V-dependent nitrogenase expression. This newly constructed strain, whose genotype was confirmed by DNA sequence analysis of genomic DNA, is designated DJ1254. A Km^R cassette was then inserted near the N-terminus coding region (located at a Mfe1 restriction enzyme site) of *vnfK* (located at the residue 33-encoding position) in DJ1254 by transformation using plasmid pDB1087 to yield strain DJ1255. Strain DJ1255 is disabled for both Mo-dependent and V-dependent nitrogenase and cannot grow in nitrogen-free media containing either Mo or V. Two separate plasmids, one carrying a poly-histidine encoding sequence (5' AATTTAAAT CAC CAC CAT CAC CAC CAT CAC CAT 3') pDB1092, or one carrying a Strep-tag encoding sequence (5' GCT AGC TGG AGC CAC CCG CAG TTC GAA AAA 3') pDB2187) located at the N-terminus Mfe1 site within the VnfK coding sequence were used to rescue the Vnf-minus phenotype to yield strains DJ1258 and DJ2253, respectively. Both strains exhibit Vnf-plus and Km-sensitive phenotypes. Correct placement of the corresponding tags was confirmed by genomic DNA sequencing of the *vnfK* coding region. Derivatives of DJ2253 that are deficient in either NifB (DJ2256) or NifE

(DJ2330) were constructed by transforming DJ2253 with plasmids that carry deletions and Km^R insertions in cloned segments of either *nifB* (pDB218) or *nifE* (pDB259).

Cell growth, and protein purification. *Azotobacter vinelandii* DJ strains expressing the V-dependent nitrogenase system were grown at 30°C in a 150-liter custom built fermenter (W. B. Moore, Inc., Easton, PA) in modified Burk medium containing 2 µM V_2O_5 as the vanadium source and 10 mM urea as a nitrogen source. Parameters for growth and cell harvesting were the same as described previously.[4] The Strep-tagged VFe proteins were purified according to a published procedure using a Strep-Tactin (IBA Lifesciences, Göttingen, Germany) column.[4] Fe protein specific for the V-dependent system was purified from strains DJ1258 or DJ2330 using a procedure similar to that for purification of Fe protein from the Mo-dependent system as described before.[5,6] The identity of purified proteins was determined by mass spectrometry as previously described.[7]

***In vitro* incubation of $VFe^{Str\Delta nifE}$ protein**. Crude extract from DJ2330 (100 g wet cells) was incubated with 10 mM sodium dithionite (DT), 50 µM V_2O_5, 2.0 mM α-ketoglutaric acid and MgATP regenerating mix, that consisted of 2.5 mM ATP, 30 mM creatine phosphate, 10 mM $MgCl_2$ and 0.1 mg/mL of creatine phosphokinase. The reaction mix was incubated at room temperature for 4h under continuous stirring under an argon atmosphere. The reaction mixture was then filtered through a 0.45 µm membrane and loaded on 2 tandem strep-tactin columns (5 mL each). Nonspecifically bound proteins were removed from the column by washing with 30 mL of Buffer A (50 mM Tris, 500 mM NaCl, 20% glycerol, 2 mM DT, pH 8.0). Elution was performed with 50 mM biotin in buffer A.

Protein activity assays. Substrate reduction assays were conducted in 9.4-mL septum-sealed serum vials with 1 mL of an assay buffer containing a MgATP regeneration system (5.0 mM ATP, 6.7 mM $MgCl_2$, 30 mM phosphocreatine, 0.2 mg/mL creatine phosphokinase, and 1.0 mg/mL BSA) and 10-12 mM DT in 100 mM MOPS at pH 7.3. The reaction vials were degassed under vacuum and the headspace was refilled with Ar for proton reduction or with N_2 for N_2 reduction and adjusted to 1 atm. The VFe^{Str} protein was then added to the reaction buffer to a final concentration of 0.1 mg/mL. Each reaction vial was preincubated in a 30 °C water bath for 2 min before the addition of Fe protein with a molar ratio to VFe^{Str} protein of ≥ 20 to start the reaction at the same temperature. The reactions lasted for 8-10 min as specified with a shaking rate of *ca.* 140 rpm before the reactions were quenched by addition of 500 µL of 400 mM EDTA at pH 8.0. The products (H_2 and NH_3) were quantified according to methods described before.[8] The detailed information about assay conditions and protein concentrations are listed in the corresponding table captions and figure legends.

EPR sample preparation. The resting state of VFe^{Str} proteins were prepared in 100 mM MOPS buffer, pH 7.3, with 150 mM NaCl and 20 mM DT, and the resting state Fe protein was prepared in turnover buffer described described below. Turnover samples prepared for EPR analyses included 200 mM MOPS buffer at pH 7.3 with a MgATP regeneration system (20 mM MgATP, 20 mM phosphocreatine, 1 mg/mL bovine serum albumin, and 0.4 mg/mL creatine phosphokinase from rabbit muscle) and 20 mM DT. VFe^{Str} protein was first added to the designated final concentration and the reaction initiated by addition of Fe protein to the

designated concentration. After the reaction was incubated at room temperature for about 20-25 s, an aliquot of ~300 µL of the reaction mixture was rapidly transferred into a 4-mm quartz EPR tube and frozen in a pentane slurry before being stored in liquid nitrogen for EPR measurement. The protein concentrations of the samples can be found in the corresponding figure legends.

The samples for EPR study of the redox properties of VFe^{Str} protein were made in 100 mM MOPS at pH 7.3 with 150 mM NaCl as the following: (1) a resting state sample of VFe^{Str} protein with 20 mM DT; (2) two methylene blue (MB, E_m = +11 mV vs SHE)[9] oxidized samples were prepared in parallel in the same buffer with a final concentration of 612 µM MB and VFe^{Str} protein and no DT . After these two reactions were incubated for 15 min at room temperature, one sample was immediately frozen in an EPR tube whereas DT was added to the other sample, giving a DT concentration of 20 mM, and then frozen. All three samples contained a final VFe^{Str} protein concentration of 50 µM.

To confirm the observed intermediate hyperfine splitting is associated with a catalytic intermediate, a pair of EPR samples under Ar turnover condition were made. One Ar turnover EPR sample was made as mentioned above with a final VFe^{Str} protein concentration of 10 µM and Fe protein concentration of 100 µM. The sample was freeze quenched after incubation for 25 sec at room temperature. The other sample was made with the same amount of the proteins and incubation time (25 sec) at room temperature before the addition of a degassed and dithionite-reduced 400 mM EDTA stock solution to bring the final protein concentrations to the same as those in the first sample and with a final EDTA concentration of 75 mM. After 300 s relaxation at room temperature,to allow for relaxation**,** the sample was frozen in a pentane slurry before being stored in liquid nitrogen for EPR measurement. Figure S5 shows the signal from the intermediate disappears during the relaxation period.

EPR spectroscopy. Continuous-wave (CW) X-band EPR spectra were recorded using a Bruker ESP-300 spectrometer with an EMX PremiumX microwave bridge and an EMX^{PLUS} standard resonator in perpendicular mode, equipped with an Oxford Instruments ESR900 continuous helium flow cryostat using VC40 flow controller for helium gas. Spectra were recorded at the following conditions: temperature, ~12 K or otherwise stated in the figure legends; microwave frequency, ~9.38 GHz; microwave power, 20 mW; modulation frequency, 100 kHz; modulation amplitude, 8.14 G; time constant, 20.48 ms. Each spectrum is the sum of five or ten scans as specified for each data set as in the figure legends, and is presented in this work after subtracting the cavity background signal recorded with an EPR tube with frozen 100 mM MOPS buffer at pH 7.3 with 150 mM NaCl. EPR simulations were performed with the EasySpin program operating in Matlab *5.2.83*

Spin quantification of EPR signals. Spin quantification of S = ½ EPR signals observed for VFe protein preparations was carried out under non-saturating conditions with Cu^{2+}-EDTA as standards according to an established procedure.[10] The concentration of a stock Cu^{2+}-EDTA solution (21.766 mM) in 100 mM MOPS buffer with 150 mM NaCl at pH 7.3 were calibrated

with an extinction coefficient of 0.094 $mM^{-1}\bullet cm^{-1}$ at 730 nm.[11,12] The Cu^{2+}-EDTA standard samples were made by sequential dilutions to final concentrations of 50 and 100 μM. EPR measurements of the samples were collected at the same microwave power, modulation amplitude, temperature, and number of scans. The baselines were corrected, when necessary, before the final integrations were done using Bruker WinEPR processing software. The g-value differences were taken into account for the final quantitative analysis.

Table S1. Specific activities of VFe proteins from the EPR samples listed in **Figure 4**.[a]

**VFe^{Str} protein**	**Substrates**		
	**Protons (1 atm Ar)**	**N_2 (1 atm) and protons**	
	nmol of H_2/min/mg	nmol of H_2/min/mg	nmol of NH_3/min/mg
Untreated	1801 ± 19	975 ± 19	350 ± 3
MB-oxidized	947 ± 8	561 ± 5	175 ± 8
DT re-reduced after MB oxidation	1005 ± 9	577 ± 12	179 ± 4

[a] All assays were performed in a MOPS buffer, pH 7.3, at 30 °C for 8 min at a molar ratio of VFe^{Str} protein to Fe protein of 1:30, and the specific activities are expressed as nmol of product per min per mg of VFe^{Str} protein as an average with standard deviation.

Figures

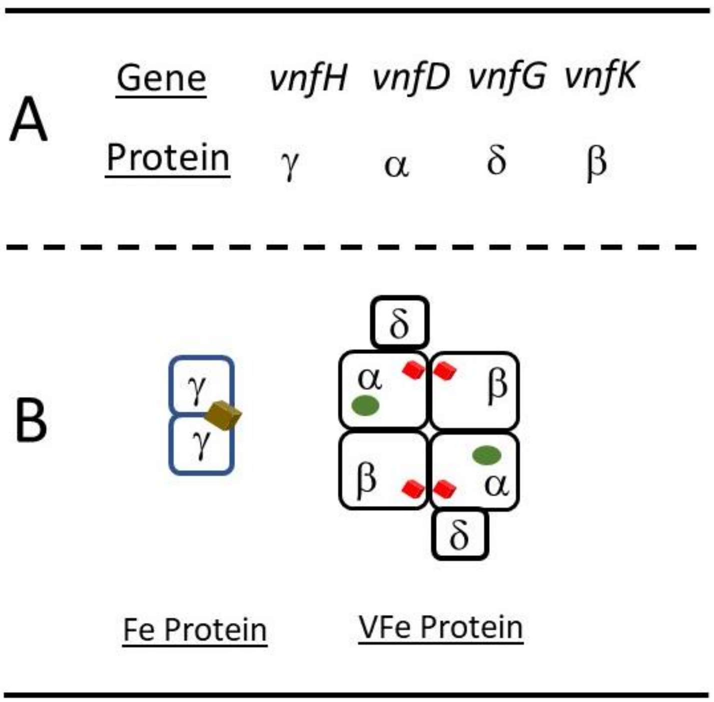

Figure S1. Schematic representation of gene and protein designations for the structural components of V-dependent nitrogenase (A) and subunit organization of the Fe protein and VFe protein catalytic partners (B). Associated metal clusters are indicated in B as [4Fe-4S] (tan cube), [8F-7S] P-cluster (tandem red cubes), and [7Fe-9S-V-C Homocitrate] FeV-cofactor (green oval). The crystal structures have been determined for Fe protein[13] and VFe protein[14] from V-dependent nitrogenase system.

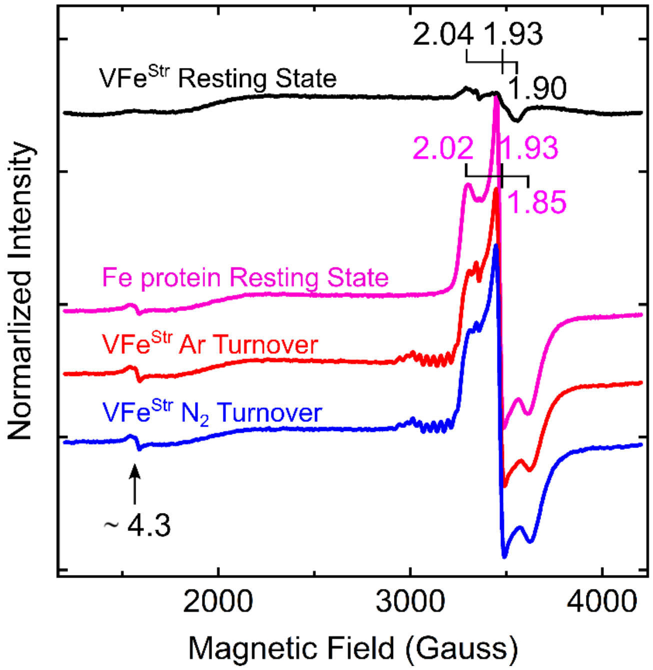

Figure S2. X-band EPR spectra of V-nitrogenase proteins. Spectra are shown in the resting states (black trace) of VFe^{Str} protein (indicated as VFe^{Str}) and Fe protein (magenta trace). Also shown are spectra for Fe protein-VFe^{Str} protein freeze-trapped during turnover under Ar (red trace) and N_2 (blue trace). The g-values of the S = ½ signals for resting state VFe^{Str} protein and Fe protein are labeled, as well as the contaminating S = 3/2 species at g ≈ 4.3 in resting state Fe protein and turnover spectra. Sample information and experimental conditions are the same as those detailed in **Figure 5**.

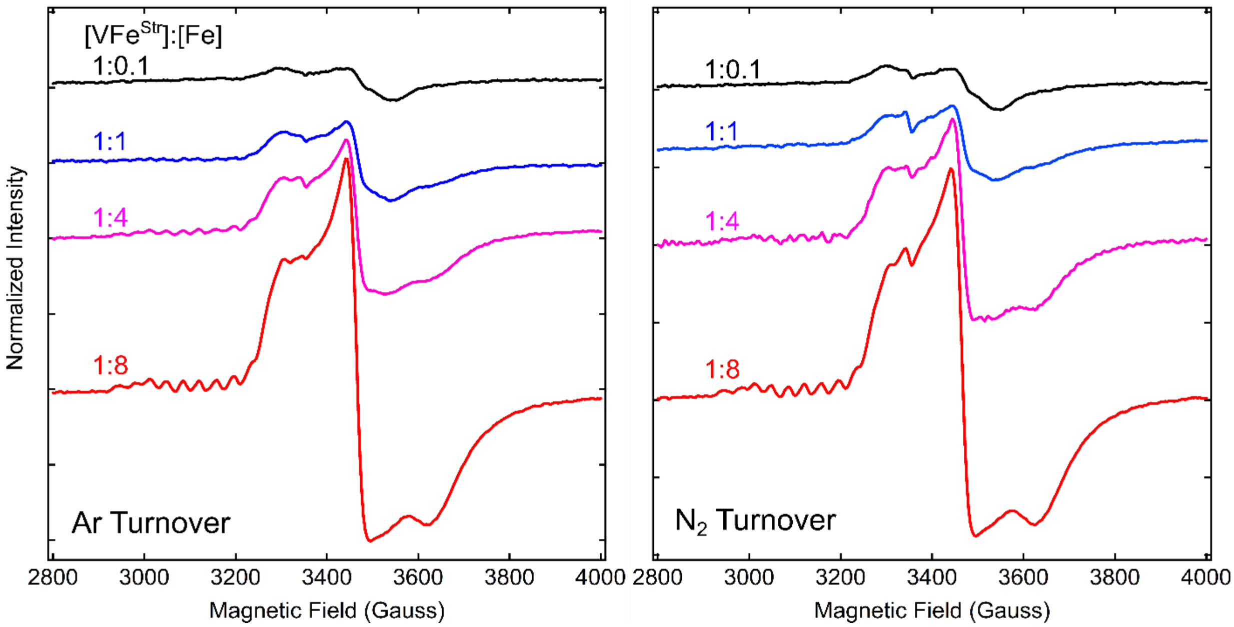

Figure S3. EPR spectra for Fe protein-VFe^{Str} protein freeze trapped during turnover under Ar (left panel) and N_2 (right panel). All samples contain 5 µM of VFe^{Str} protein (indicated as VFe^{Str}) with different concentrations of Fe protein (0.5 µM for 1:0.1, 5 µM for 1:1, 20 µM for 1:4, and 40 µM for 1:8 traces). All spectra were recorded at 12 K with a microwave power of 20 mW applied. Each trace is the sum of 10 scans.

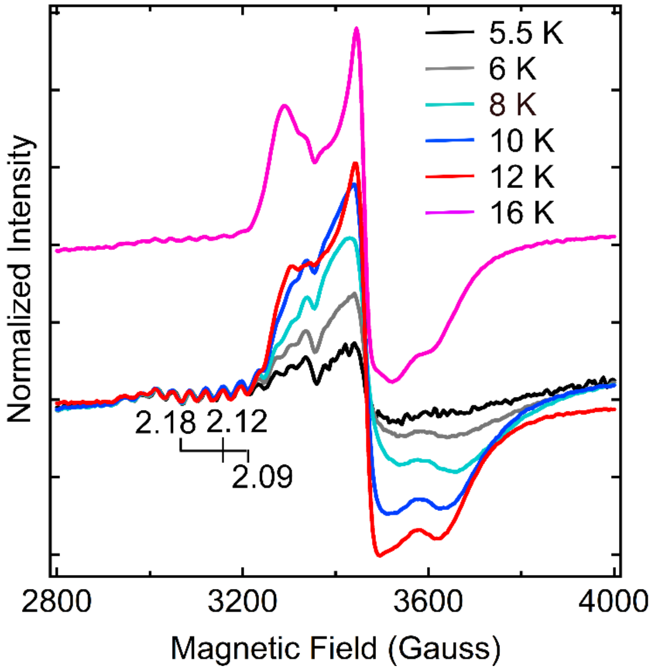

Figure S4. EPR spectra for freeze-trapped V-nitrogenase during turnover under Ar with 5 μM of VFe^{Str} protein and 40 μM of Fe protein recorded at different temperatures. The g-values (g = 2.18, 2.12, and 2.09) for the S = ½ signal with ^{51}V (I = 7/2) hyperfine splitting were indicated. The EPR spectra were recorded with an applied microwave power of 20 mW with varied temperatures from 5.5 K up to 16 K. To better visualize the intensity decrease of the S = ½ signal with ^{51}V (I = 7/2) hyperfine splitting upon the temperature increase to 16 K, the spectrum recorded at 16 K was plotted separately.

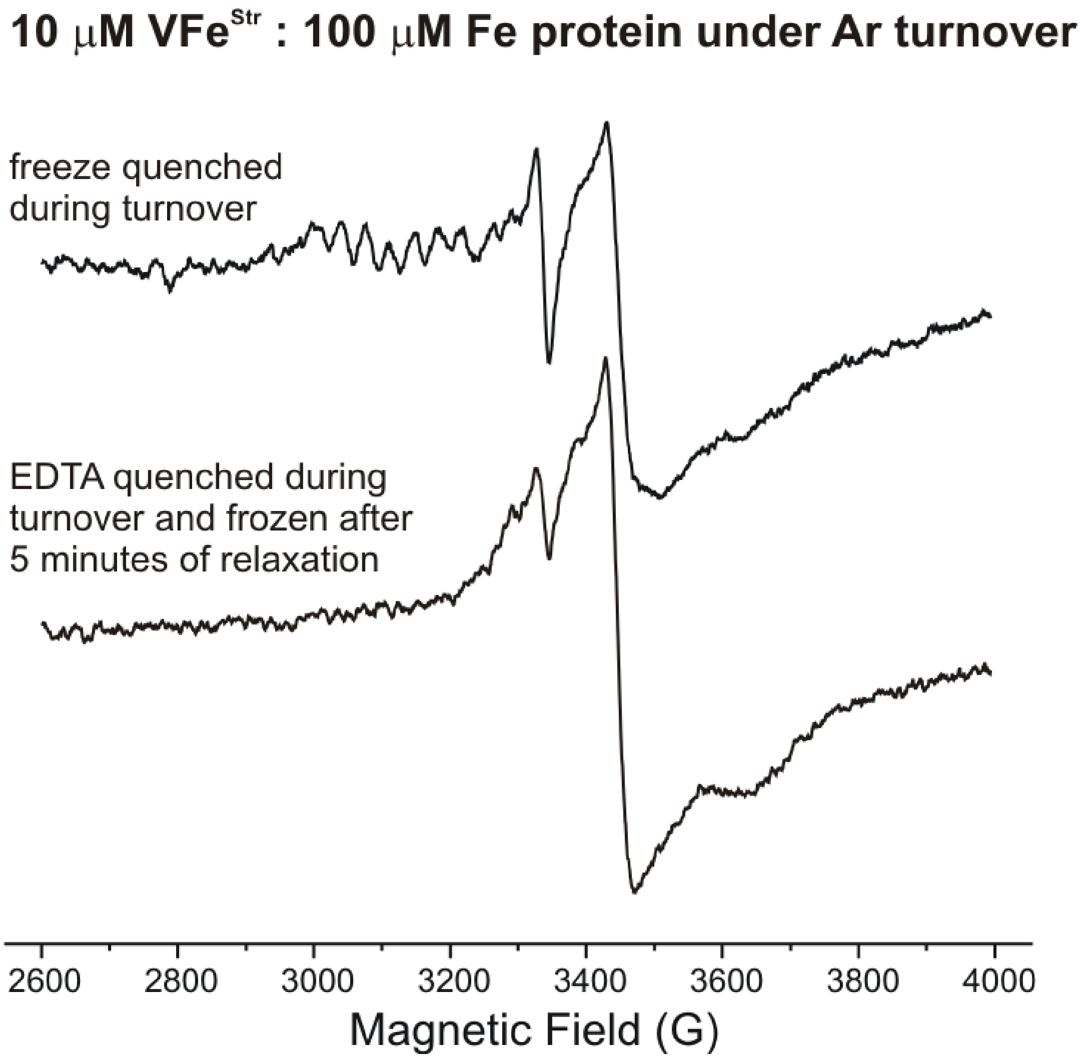

Figure S5. X-band EPR spectra of 10 μM VFe^{Str} protein:100 μM Fe protein under Ar turnover (upper) freeze quenched after 25 seconds of incubation and (lower) frozen after 25 seconds of incubation followed by reaction quench by EDTA addition and relaxation for 5 minutes. The spectra were recorded at 3.8 K; each trace is the sum of 10 scans.

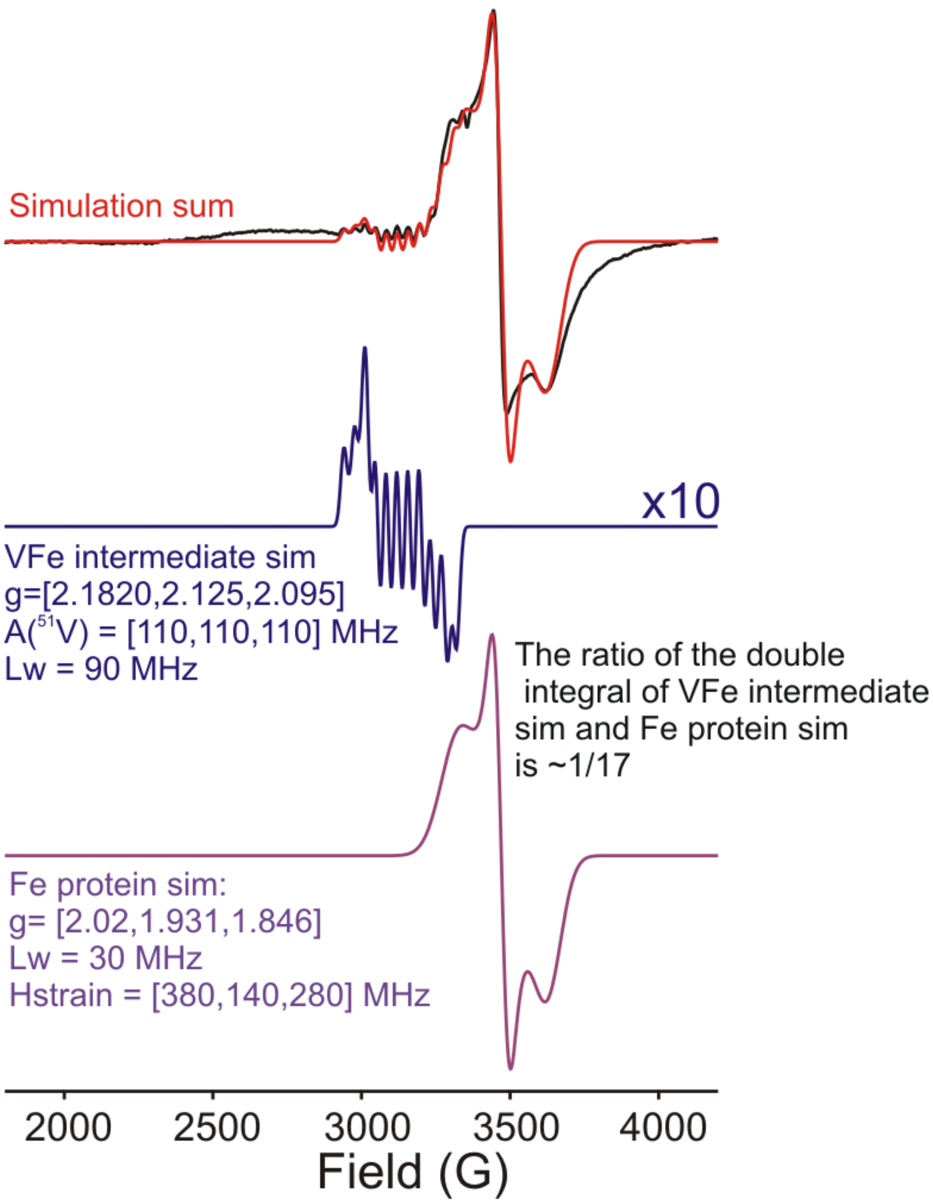

Figure S6. Simulations of EPR spectra for freeze-trapped V-dependent nitrogenase during turnover under Ar with 5 µM VFe^{Str} protein and 40 µM Fe protein recorded at 12 K. The simulation parameters are given in the figure.

References

1 A. C. Robinson, B. K. Burgess and D. R. Dean, *J. Bacteriol.*, 1986, **166**, 180–186.
2 R. Premakumar, S. Jacobitz, S. C. Ricke and P. E. Bishop, *J. Bacteriol.*, 1996, **178**, 691–696.
3 J. Noar, T. Loveless, J. L. Navarro-Herrero, J. W. Olson and J. M. Bruno-Bárcena, *Appl. Environ. Microbiol.*, 2015, **81**, 4507–4516.
4 E. Jiménez-Vicente, J. S. Martin Del Campo, Z.-Y. Yang, V. L. Cash, D. R. Dean and L. C. Seefeldt, in *Methods in Enzymology*, ed. F. Armstrong, Academic Press, 2018, vol. 613, pp. 231–255.
5 B. K. Burgess, D. B. Jacobs and E. I. Stiefel, *Biochim. Biophys. Acta*, 1980, **614**, 196–209.
6 J. W. Peters, K. Fisher and D. R. Dean, *J. Biol. Chem.*, 1994, **269**, 28076–28083.
7 E. Jimenez-Vicente, Z.-Y. Yang, J. S. Martin del Campo, V. L. Cash, L. C. Seefeldt and D. R. Dean, *J. Biol. Chem.*, 2019, **294**, 6204–6213.
8 B. M. Barney, R. Y. Igarashi, P. C. Dos Santos, D. R. Dean and L. C. Seefeldt, *J. Biol. Chem.*, 2004, **279**, 53621–53624.
9 G. D. Watt and Z. Wang, *Biochemistry*, 1986, **25**, 5196–5202.
10 R. Aasa and T. Vänngård, *J. Magn. Reson.*, 1975, **19**, 308–315.
11 S. Georgieva, T. Nedeltcheva, L. Vladimirova and A. Stoyanova-Ivanova, *Cent. Eur. J. Chem.*, 2013, **11**, 381–387.
12 T. Nedeltcheva, *Anal. Chim. Acta*, 1995, **312**, 223–226.
13 M. Rohde, C. Trncik, D. Sippel, S. Gerhardt and O. Einsle, *J. Biol. Inorg. Chem.*, 2018, **23**, 1049–1056.
14 D. Sippel and O. Einsle, *Nat. Chem. Biol.*, 2017, **13**, 956–960.
